# The might of light for revealing neuropsychiatric mechanisms

**DOI:** 10.1038/s41386-024-01974-4

**Published:** 2024-09-12

**Authors:** Kutlu Kaya, Hilary P. Blumberg

**Affiliations:** 1 Department of Psychiatry, Yale School of Medicine, New Haven, CT USA; 2 Department of Radiology and Biomedical Imaging, Yale School of Medicine, New Haven, CT USA; 3 Child Study Center, Yale School of Medicine, New Haven, CT USA

**Keywords:** Psychiatric disorders, Neuroscience

Human in vivo optical neuroimaging has strong potential to advance neuropsychiatric disease elucidation and treatment. Akin to functional magnetic resonance imaging (fMRI) for a proxy for brain hemodynamics and activity, functional near-infrared spectroscopy (fNIRS) systems apply two near-infrared wavelengths of light and read out reflections from the head to quantify real-time changes in hemoglobin oxygenation. Moreover, fNIRS measures are non-invasive, achieved using wearable devices, and have less movement sensitivity, facilitating feasibility and patient acceptability, and permitting measures in movement-prone populations, including children or individuals with movement disorders. Portable fNIRS devices permit study in ecologically salient environments, elucidating responses of brain systems subserving social processes in “real-world” settings. For example, fNIRS recently showed sensitivity in detecting differences in regional brain responses to in-person, compared to videoconferenced, face processing [1]. As social dysfunction is central to suffering and disability of psychiatric conditions, study in real-world settings may provide critical new information about disorder mechanisms that prior were elusive.

FNIRS’ feasibility also makes it amenable to repeated measurements to follow treatment response or disease progression. Temporal patterns of brain activity and related behaviors are increasingly thought to hold essential information about the pathophysiology of neuropsychiatric conditions [2] and can provide early indicators of worsening to prevent progression and improve prognosis.

**Fig. 1:**
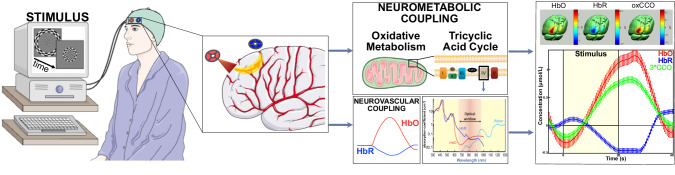
Human in vivo Broadband Near-Infrared Spectroscopy (bNIRS). Broadband near-infrared spectroscopy (bNIRS) detects light attenuation in the optical spectrum of 650–900 nm to quantify regional concentration changes in oxy-hemoglobin (HbO), deoxy-hemoglobin (HbR), and mitochondrial complex IV cytochrome-c-oxidase oxidative states (oxCCO) of the vascular bed under optical sensors (optodes) during a particular task. The optodes are arranged over the brain regions of interest within the cap. Flexible fiber optic cables (not shown) transmit the signals to computers for data processing. The right images are adapted from Bale et al. [3] and are courtesy of Ilias Tachtsidis, PhD, University College London, UK. The Figure was drawn in part using images from Servier Medical Art by Servier, which is licensed under a Creative Commons Attribution 3.0 Unported License.

As an extension of fNIRS, Tachtsidis et al developed broadband NIRS, bNIRS, methods that allow extended NIR light spectrum acquisition (hundreds of NIR wavelengths) [3, 4]. BNIRS can therefore simultaneously provide fNIRS measures of hemodynamics and measures such as ones reflecting mitochondrial functioning, enabling study of relationships between regional brain dysfunction and mitochondrial pathology (Fig. 1). This is important and timely given recent research increasingly revealing the importance of mitochondrial mechanisms in neuropsychiatric conditions [5]. Prior in vivo neuroimaging research to understand mitochondrial dysfunction had typically utilized ^1^H or ^31^P magnetic resonance spectroscopy (MRS), which can provide lactate and phosphorylation pathway measures reflecting oxidative metabolism; however, the equipment size and immobility limit its feasible use. Moreover, bNIRS can provide measurements related to mitochondrial complex IV cytochrome-c-oxidase oxidative states (oxCCO), shown important in neuropsychiatric disorders and increasingly considered a future diagnostic biomarker and treatment target. Initial support is provided by a 4-wavelength NIRS study conducted among 7 individuals with major depressive disorder and 6 with bipolar disorder (BD) that showed lower prefrontal oxCCO in BD inversely associated with depression severity [6]. Studies of neonatal or traumatic brain injury support the utility of bNIRS across a variety of neuropsychiatric conditions and to reveal hemodynamic-oxidative decoupling [3, 4].

NIRS measures are limited to the brain’s outer cortex, given light’s penetration depth. Recent multichannel NIRS methods have expanded brain coverage and increased spatial resolution. Especially promising for mechanistic study are recent advances in integrating NIRS with other imaging methods that can provide complementary data, such as higher temporal resolution of simultaneous electroencephalography, greater spatial resolution and depth allowing for study of cortical-subcortical connectivity of fMRI, and mitochondria-related measures of MRS.

In summary, NIRS is an important technology for neuropsychiatry’s future to improve disorder understanding, diagnosis and treatment.
