# Correction: The might of light for revealing neuropsychiatric mechanisms

**DOI:** 10.1038/s41386-024-02032-9

**Published:** 2024-12-09

**Authors:** Kutlu Kaya, Hilary P. Blumberg

**Affiliations:** 1 Department of Psychiatry, Yale School of Medicine, New Haven, CT USA; 2 Department of Radiology and Biomedical Imaging, Yale School of Medicine, New Haven, CT USA; 3 Child Study Center, Yale School of Medicine, New Haven, CT USA

**Keywords:** Psychiatric disorders, Neuroscience

Correction to: *Neuropsychopharmacology* 10.1038/s41386-024-01974-4, published online 12 September 2024

The article “The might of light for revealing neuropsychiatric mechanisms”, written by Kutlu Kaya and Hilary P. Blumberg, was originally published Online First without Open Access. After publication in volume 50, issue 1, pages 335-336, the author decided to opt for Open Choice and to make the article an Open Access publication. Therefore, the copyright of the article has been changed to © The Author(s) 2024 and the article is forthwith distributed under the terms of the Creative Commons Attribution 4.0 International License, which permits the use, sharing, adaptation, distribution, and reproduction in any medium or format, as long as you give appropriate credit to the original author(s) and the source, provide a link to the Creative Commons license, and indicate if changes were made. The images or other third-party material in this article are included in the article’s Creative Commons license unless indicated otherwise in a credit line to the material. If material is not included in the article’s Creative Commons license and your intended use is not permitted by statutory regulation or exceeds the permitted use, you will need to obtain permission directly from the copyright holder. To view a copy of this license, visit http://creativecommons.org/ licenses/by/4.0/.

